# Evaluation of a Year During the COVID-19 Pandemic in a Private Healthcare Facility: Retrospective Cross-Sectional Study

**DOI:** 10.7759/cureus.23236

**Published:** 2022-03-16

**Authors:** Burçin Budakoğlu, Tonguç Sugüneş, Ferit Aslan, Şule Öner, Aydın Nadir

**Affiliations:** 1 Department of Internal Medicine, Division of Medical Oncology, Medical Park Ankara Hospital, Ankara, TUR; 2 Department of General Surgery, Medical Park Ankara Hospital, Ankara, TUR; 3 Department of İnternal Medicine, Division of Medical Oncology, Medical Park Ankara Hospital, Ankara, TUR; 4 Department of Infection Control, Medical Park Ankara Hospital, Ankara, TUR; 5 Department of Thoracic Surgery, İstinye University Faculty of Medicine, Ankara, TUR

**Keywords:** healthcare service delivery, manpower shortage, healthcare workers, healthcare, covid 19, workdays, hospital economy

## Abstract

Aims and Objectives: To examine the impact of the workforce crisis on healthcare service delivery for a year during the coronavirus disease 2019 (COVID-19) pandemic on healthcare service delivery and hospital economy in a healthcare facility.

Materials and Methods: An examination was conducted of employees who were issued with a report for incapacity to work due to the pandemic between March 2020 and March 2021. A record was made of the employees' ages, genders, fields of work and lost workdays. The employees were classified as physicians, nursing services, guest services, hotel services, and support services. Diagnoses were made of the novel severe acute respiratory syndrome coronavirus 2 (SARS-CoV-2), based on computed tomography (CT) and polymerase chain reaction (PCR) test results. Employees diagnosed with SARS-CoV-2 infection were put into isolation in the hospital during the first months of the pandemic, and treatment was initiated in accordance with the protocols. By contrast, during the last eight months, our personnel who were not indicated for hospitalization completed their treatment in a home isolation setting. According to the Turkish Ministry of Health COVID-19 (SARS-CoV-2 Infection) Scientific Advisory Board Study Guide, the isolation period was initially 14 days, before being reduced to 7-10 days, in line with the revised guide. Employees with at least one negative PCR test result following isolation were allowed to return to work.

Results: The study included 575 individuals who were employed at our hospital between March 2020, when the first case was identified in Turkey, and March 2021. Among these employees, 257 were issued with a report for incapacity to work due to COVID-19. Of these, 239 had a positive PCR test result. There were 11 individuals who just had symptoms and/or positive CT findings but a negative PCR test result. There were only seven individuals who were put into isolation due to high-risk contact. The combined lost workdays of the employees totaled 3792. The highest lost workday rate (52%) was in nursing services (1973 days, n = 126). There was no mortality.

Conclusion: Lost workdays due to the pandemic in the midsize healthcare facility severely affected the morale and motivation of both the diagnosed and the remaining employees. Hospital administrations also had difficulties in maintaining the quality and continuity of the services provided.

## Introduction

Issues, such as unemployment and lost workdays due to the coronavirus disease 2019 (COVID-19) pandemic, have been emphasized in the discussions across various platforms particularly with respect to several fields such as hospitality, agriculture, and tourism. However, the effects on the healthcare industry or sector and their employees have been ignored. The pandemic has created workforce crisis in hospitals all over the world giving rise to a lack of staff and technicians to manage the sudden increase in patient inflow in a difficult situation due to employment leave, the initial inadequacy of personal protective equipment (PPE), supply chain failures, and reduced hospital revenues [1-3]. Health professionals were exposed to severe acute respiratory syndrome coronavirus 2 (SARS-CoV-2) infection and had to isolate themselves, which was the major factor resulting in an inadequate workforce

In recent years, the need for human resources in the labor market, especially in the industrial and agricultural sectors, has gradually decreased due to the developing technology. Although the need for human resources has also decreased in the service sector, the need for a workforce in the healthcare sector has increased during the pandemic [2,4]. Together with the priority of staying healthy, healthcare workers continue to operate with quality care and efficient treatment. Since patient satisfaction is a determining factor in competition among private healthcare providers, it is important to maintain the service delivery without causing any interruption, or reputational risk. It is well-known that patient-employee satisfaction and brand equity in institutions can be negatively affected by reduced service quality due to employee-related lost workdays.

According to reports by the World Health Organization (WHO), healthcare workers accounted for 3-20% of COVID-19 cases in the early days of the pandemic [5,6]. These numbers started to decrease following the introduction of the vaccines. The aim of this study is to examine the impact of lost workdays due to the COVID-19 pandemic in a private hospital, its effect on the quality and efficiency of the services provided, and the impact of workforce crisis on the economy of the healthcare sector.

## Materials and methods

The study examined employees who were diagnosed with COVID-19 and those who were treated and put into isolation due to high-risk contact between March 2020 and March 2021. This study was designed as cross-sectional and retrospective. Being a hospital employee during the pandemic process was considered as the criterion for inclusion in this study. Demographics of the eligible patients including age, gender, and profession were recorded and lost workdays were calculated. An evaluation was made of 575 hospital staff including physicians, nursing services, guest services (secretaries and physician assistants), hotel management and cleaning services, and support services (technical service, biomedical department, information processing, corporate marketing, corporate billing, human resources, and purchasing department).

The employees’ reports for incapacity to work during the pandemic were determined according to Turkish Ministry of Health Scientific Advisory Board guidelines. The COVID-19 diagnoses were based on computerized tomography (CT) and polymerase chain reaction (PCR) test results. Employees who were in close contact at the hospital and at home were also included in the study. Employees diagnosed with COVID-19 were treated as inpatients during the first months of the pandemic, in accordance with the guidelines published by the Ministry of Health, and during the last eight months, those who were not indicated for hospitalization were put into home isolation until recovery. The initial isolation period of 14 days was eventually reduced to 7-10 days in accordance with the Ministry of Health guidelines (7). Employees with at least one negative PCR test result following isolation were allowed to return to work.

The lost workdays of employees who were issued with a report for incapacity to work were calculated using the severity rate formula for accidents at work. It was calculated as the number of lost workdays per 1,000 working days

## Results

The study comprised 575 individuals who worked at our hospital between March 2020, when the first case was identified in Turkey, and March 2021. Follow-up was made of 94 (16.3%) physicians, 226 (39.5%) employees from nursing services, 90 (15.6%) from guest services, 105 (18.2%) from hotel management and cleaning services, and 60 (10.4) from support services (Table 1).

**Table 1:** Numbers of PCR, CT, and workdays of COVID-19 patients. PCR: polymerase chain reaction; CT: computed tomography; COVID-19: coronavirus disease 2019

DEPARTMENT	PERSONNEL NUMBER (N)	PCR (+) (N)	PCR (-) (N)	CT (+) / SYMPTOMS (N)	CONTACT (N)	PERSONNEL WITH TO COVID-19 EXPOSURE (N)	TOTAL NUMBER OF SICK REPORTS (N)	TOTAL NUMBER OF SICK DAYS (N)	DEPARTMENT OF PERSONNEL WITH TO COVID-19 EXPOSURE (%)
PHYSICIAN	94	28	9	6	3	37	37	484	39.36%
DEPARTMENT OF NURSING	226	120	6	5	1	126	126	1973	55.00%
DEPARTMENT OF GUEST SERVICES	90	32	1	0	1	33	33	485	36.17%
DEPARTMENT OF HOTEL MANAGEMENT AND CLEANING SERVICES	105	39	0	0	0	39	39	578	37.14%
SUPPORT SERVICES	60	20	2	0	2	22	22	272	36.66%
TOTAL(N)	575	239	18	11	7	257	257	3792	44.50%

Among the 257 employees who were issued with a report for incapacity to work, there were 181 women and 76 men. The youngest and oldest patients were 19 and 60 years old, respectively. The rate of inpatients was 22.17% (n = 57) while the rate of outpatients was 77.82% (n = 200). Only one of the inpatients was followed up in the intensive care unit. None of the patients needed mechanical ventilation. All other patients were followed up in the ward. Reports for incapacity to work were issued to 37 (39%) physicians, 126 (55.7%) employees from nursing services, 33 (36.6%) from guest services, 39 (37%) from hotel management and cleaning services, and 22 (36.6%) from support services (Table 1). In the period between October to November 2020, the total number of people who were on sick leave during the same period reached 68 (12%).

Among the study participants, there were 239 (41.5%) patients with positive PCR test results. A total of 11 individuals were detected with positive CT findings and/or symptoms but negative PCR test results. Only seven individuals were put into isolation due to contact. Those who had contact and were PCR negative in the first test but PCR positive in the second and/or third test were excluded from the contact group. These individuals were included in the PCR-positive group. In this study, six people were diagnosed with COVID-19 for the second time; of these, four were diagnosed with COVID-19 after vaccination and two before vaccination. The highest rate of COVID-19 diagnosis was among employees from patient care services (52%, n = 126). Of the diagnosed patient care workers, 15.8% (n = 20) were working in intensive care units and 84.2% (n = 106) in clinics and outpatient clinics.

The number of sick days of the employees ranged from seven to 41 days, with a mean of 14.7 days. The combined lost workdays of all employees were 3792 days. The direct incapacity rate was 44.5% (575/257). The severity rate for accidents at work was approximately 21 days (21/1,000 days). This rate was 5.5 (5.5/1000) days in the first quarter of 2021. The highest number of lost workdays was in nursing services with 1973 days (52%), followed by hospital management and cleaning services with 578 (15%) days (Table 1).

## Discussion

The emergence of the pandemic has deeply affected the healthcare sector as well as social determinants of health such as education, employment, social activities, working life, transportation, and trade in almost every country [1,2,7,8]. There are countries in Europe where healthcare sectors have collapsed and even come to a halt. In countries with inadequate infrastructure, intensive care units had to choose from among their patients (such as young age, without comorbidity). The delivery of healthcare services under intense pressure has challenged directors and clinicians in providing quality care to patients. In fact, some hospitals had to take measures including the closure of wards when almost all the clinicians were infected. 

Pandemics pose serious threats to public health by causing a large number of deaths, as well as physical and mental health problems [3-5,9,10]. Healthcare workers were faced with many different problems, in addition to their normal working conditions. Working at an intense pace, under stress, and having concerns for their own health and the health of their families, made the service delivery obligation challenging. Several uncertainties, especially in the early days of the pandemic, had negative physical and psychological impacts on healthcare workers. In their study, Doherty et al. reported that 77% of physicians experienced burnout syndrome during the first 11 months of the pandemic. In the same study, approximately two-thirds of the participants reported a negative impact on their mental health [3]. Studies have shown that the anxiety levels of the healthcare workers had increased when their colleagues become sick or died, leaving them increasingly subdued [8]. Due to the need for social isolation, in order to continue where life left off, it has become important to continue with activities using the opportunities provided by technology. Digitalization is also performing a crucial role in the healthcare sector; approaches such as teleconferencing, and telemedicine have emerged, and homecare services have been strengthened [2,11,12].

In 2020, when the COVID-19 pandemic was dominant, reports estimated that global economic growth had decreased from around 3% to 2.4%, triggering an economic loss of approximately 3.5 trillion dollars worldwide [2,13,14]. Estimates suggested that at the international level the pandemic had brought about shrinkage of 13-32% [5]. The unpredictable course of the pandemic, together with economic uncertainty, made decision-making difficult for the investors. The COVID-19 crisis had also deeply affected health economics. According to economists, hospitals have experienced an economic shock that will result in a U-shaped recovery, which can be described as "reduced economic growth, then a very gradual recovery, only slowly returning to pre-crisis levels" [2]. Public hospitals suffered relatively less from this crisis due to government support. Private hospitals, on the other hand, have experienced a deeper crisis, especially due to supply chain failures, an absent workforce, and a reduction in elective surgeries.

Our hospital management provided our staff with 3540 minutes of COVID-19 pandemic training, based on the best clinical data available and the Ministry of Health guidelines in this study. The training was repeated in line with new information. A pandemic board was established, comprising a pulmonologist, an infectious diseases specialist, an anesthesiologist, an occupational physician, and an infection control nurse. Updated information was regularly shared with employees. These practices were found to reduce anxiety and have a positive effect on the quality of service provided. The severity rate for accidents at work was 21 in the first period of our study, decreasing to 5.5 in the last four-month period. We consider that the following were effective in decreasing this rate: understanding the characteristics of the pathogen causing the infection, having easy access to personal protective equipment, commencing the vaccinations, attaching importance to systematic training and practices, and having supervision and guidelines. 

 When occupations were categorized into four risk groups during a pandemic, healthcare workers were considered to be the highest occupational risk group. Reports have stated that healthcare workers accounted for 3.8% of infection cases in China. More recent reports stated that the rate has reached up to 29%. Further reports claimed that 50% of the initial cases in Singapore and 10% in Italy were healthcare workers. This rate was reported to have reached 12% in the USA and 38% in Spain. In April, this rate was reported to have reached 6.8% in Turkey [4-6,14]. These are the data on the early days of the pandemic. Until November 2020, 4% of healthcare workers were infected. In the same period, 215 healthcare workers died [7]. In our hospital, this rate was very high for a one-year period, and 44.5% (n = 257) of 577 employees were diagnosed with COVID-19. Even so, none of our employees died. We believe that this was because of the frequent and high number of tests, especially in the early days of the pandemic, whether or not there were symptoms. 

To define a condition as an occupational disease, it must be caused by a recurring reason, due to the nature of the work, the occupational activity of the employee, or by how the work activity is conducted. The SARS-Cov-2 infection primarily affects the lungs in the early and later stages of the disease. Late pulmonary fibrosis becomes overt over time. Studies identified gas exchange worsening in 51% and decreased total lung capacity in 25% of patients at discharge, who were previously diagnosed with COVID-19 and hospitalized. It was reported that 62% of these patients had CT findings of fibrosis. It was further reported that 10% of the patients had permanent damage to the lungs [15-17]. Therefore, academia should discuss whether the condition is an occupational disease when employees, such as physicians, nurses, and cleaning staff who work in the hospital are infected in the hospital environment.

The limitation of our study was the lack of information about the hospital’s exact economic loss. The decrease in the number of elective surgeries and the number of patients presenting to the outpatient clinic, as well as the increase in procurement costs, have generated a serious economic loss for hospitals. Since the beginning of the pandemic, the frequent use of expensive diagnostic tests, the high cost of medical treatment, the planning of inpatient treatment, the need to close income-generating clinics, and the cost of vaccination have adversely affected private healthcare facilities to a certain extent.

## Conclusions

One of the most important gains of the outbreak has been to learn that it is necessary for countries to allocate funding for preventive and social medicine and to draw attention to the this public health issue. The foundations of the public healthcare system should be strengthened with a strong infrastructure that should be prioritized as a social policy, for the wellbeing of everyone in the society, contributing to the public’s awareness of wellbeing and one in which each individual can be easily accessed and followed up. There is a need to develop fast and coordinated policies at national and global levels in order to limit the direct impact of the SARS-Cov-2 virus on employees and their families, as well as mitigating its effects on the hospital economy. Senior executives who are responsible for managing activities are expected to design business models that are compatible with the pandemic, able to anticipate possible risks, can provide job security, and make employees aware of this.
